# Global view of cognate kinase activation by the human pyruvate dehydrogenase complex

**DOI:** 10.1038/srep42760

**Published:** 2017-02-23

**Authors:** Elena L. Guevara, Luying Yang, Barbara Birkaya, Jieyu Zhou, Natalia S. Nemeria, Mulchand S. Patel, Frank Jordan

**Affiliations:** 1 Department of Chemistry, Rutgers, The State University of New Jersey, Newark, New Jersey 07102, USA; 2 Department of Biochemistry, Jacob School of Medicine and Biomedical Sciences, University at Buffalo, The State University of New York, Buffalo, New York 14214, USA.

## Abstract

The human pyruvate dehydrogenase complex (PDC) comprises four multidomain components, E1, E3, E2 and an E3-binding protein (E3BP), the latter two forming the core as E2·E3BP sub-complex. Pyruvate flux through PDC is regulated via phosphorylation (inactivation) at E1 by four PDC kinases (PDKs), and reactivation by two PDC phosphatases. Up-regulation of PDK isoform gene expression is reported in several forms of cancer, while PDKs may be further activated by PDC by binding to the E2·E3BP core. Hence, the PDK: E2·E3BP interaction provides new therapeutic targets. We carried out both functional kinetic and thermodynamic studies to demonstrate significant differences in the activation of PDK isoforms by binding to the E2·E3BP core: (i) PDK2 needs no activation by E2·E3BP for efficient functioning, while PDK4 was the least effective of the four isoforms, and could not be activated by E2·E3BP. Hence, development of inhibitors to the interaction of PDK2 and PDK4 with E2·E3BP is not promising; (ii) Design of inhibitors to interfere with interaction of E2·E3BP with PDK1 and PDK3 is promising. PDK3 needs E2·E3BP core for activation, an activation best achieved by synergistic combination of E2-derived catalytic domain and tridomain.

The human pyruvate dehydrogenase complex (**PDC**) belongs to the super-family of the 2-oxo acid dehydrogenase multienzyme complexes which occupy key positions in the mitochondrial oxidation for energy production, and in the oxidation of the branched-chain amino acids^1^,^2^,^3^. The human (or mammalian) PDC is composed of multiple copies of three principal catalytic components: 20–30 copies of the thiamin diphosphate (**ThDP**)-dependent pyruvate dehydrogenase (**E1**, an α_2_β_2_-heterotetramer), 48 copies of the dihydrolipoamideacetyltransferase (**E2**), 12 copies of the FAD/NAD^+^ dependent dihydrolipoamide dehydrogenase (**E3**) and two regulatory enzymes, pyruvate dehydrogenase kinase (**PDK**, four isozymes)^3^,^4^,^5^,^6^,^7^,^8^ and pyruvate dehydrogenase phosphatase (**PDP**, two isozymes)^9^,^10^,^11^. In addition, there is in human PDC an E3-binding protein (**E3BP**) whose role appears to be communication between the E2 and E3 components. According to a “substitution” model the 48 copies of E2 and 12 copies of E3BP form the core of the human PDC, to which the peripheral components E1 and E3, PDKs, and PDPs are bound noncovalently^5^,^12^. The PDC catalyzes the oxidative decarboxylation of pyruvate with the formation of acetyl-Coenzyme A (acetyl-CoA) and NADH (H^+^) according to equation (1) and SI Fig. S1.


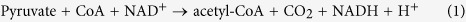


The E2 component has a multi-domain structure (Fig. 1), comprising from the N-terminal end: two tandem lipoyl domains, the outer (**L1**) and the inner lipoyldomain (**L2**) approximately 9 kDa each, a peripheral subunit-binding domain (**PSBD**, or **S**, 4 kDa) and the acetyltransferase or catalytic domain (**C**) (28 kDa), all separated by 25–30 amino acid-long flexible linkers. The E3BP is composed of three linker-connected domains, similar but not identical to those in E2, a single lipoyl domain (**L3**), a variant of S, called **S’** to which E3 binds, and a catalytic domain **C’** that, unlike **C**, is incompetent to produce acetyl-CoA.

The flux of pyruvate through PDC is tightly regulated by reversible phosphorylation of E1 at one of three serine residues, involving the PDKs and PDPs^3^,^9^,^13^,^14^. The three serines in E1 are phosphorylated *in vivo* at different rates and with different specificities by the four PDKs^15^,^16^,^17^. Site 1 is preferentially phosphorylated, and sites 2 and 3 are sequentially phosphorylated^17^,^18^. Starvation and diabetes induce PDK2 and PDK4 activity in different tissues, inducing phosphorylation and inactivation of PDC^19^,^20^,^21^,^22^. PDC is also implicated to play a role in neurodegenerative diseases, obesity, and other diseases^23^,^24^,^25^. More recently, PDC has been identified as a target for regulating glucose oxidation in cancer cells leading to the Warburg effect (aerobic glycolysis)^26^,^27^,^28^,^29^,^30^,^31^,^32^,^33^,^34^, where the pyruvate is converted to lactate, partially because of up-regulation of gene expression of PDK1^31^,^35^,^36^, PDK2^37^, and PDK3^15^,^38^,^39^. Recent studies have also revealed that targeting of PDK could serve as a novel therapeutic approach in oncology. Among known inhibitors of PDKs, the glucose-lowering compound AZD7545 (AstraZeneca)^40^, a mimic of dihydrolipoamide, exhibited efficient inhibition of PDK2 and PDK1 activities, but not of PDK4^41^,^42^. Also, the secondary amides SDZ048–619 and their substituted analogues displayed efficient inhibition of PDKs, but these compounds did not lower glucose levels in diabetic animal models^43^.

It was reported that the PDKs are activated upon binding to the E2·E3BP core: (a) The PDK2 was activated upon binding to E2^44^, or to the L2S didomain, but not to the isolated L2^45^; (b) The PDK3 was activated on binding to E2, and to the isolated L2 equally well^10^,^44^,^46^,^47^. The structure of the PDK3-L2 complex was solved in an attempt to explain the mechanism of PDK activation^47^.

The availability of individually expressed PDK1 and PDK2, E2·E3BP core and its derived domains (L1, L2S, L1L2S, L3S’) enabled us to carry out H/D exchange mass spectrometric and NMR experiments to study the interactions of the E2·E3BP core with PDK1 and PDK2 in detail^48^. It became evident that there are domains on E2, and on E3BP, in addition to the L2, that interact with PDKs^48^. Prompted by those results, we undertook this study to provide a global understanding of the role of the individual domains of the E2·E3BP core on the activation of all four PDK isoforms. For the first time, we carried out both functional kinetic and thermodynamic experiments to determine which of the domains of E2⋅E3BP core each PDK isoform recruits to be activated. We developed two new tools to achieve these goals: (a) Monitoring the time course of PDC inactivation by a particular PDK isoform according to the NADH assay of the overall PDC reaction; (b) Determination of the binding affinity between the four PDK isoforms and all E2·E3BP-derived domains (labeled with a site-specifically introduced fluorophore). Results of these studies significantly alter our understanding of PDK activation by the E2⋅E3BP core. The significant differences in the functional activation and binding isotherms among the four PDK isoforms by PDC, suggest that interaction of PDC with PDK1 and PDK3, but not with PDK2 and PDK4, could be targets for development of isozyme-specific inhibitors.

## Results

### New insight into the role of the E2·E3BP core and its derived domains on activation of four PDK isoforms as reflected by PDC inactivation kinetics

To study the activation of the four PDK isoforms by E2·E3BP core and its derived domains (L1, L2S, L1L2S, L3S’), in this paper we relied on activity measurement (NADH production) by the assembled PDC, rather than on an assay of PDK activity by incorporation of ^32^P from [γ-^32^P]ATP into E1, as reported in the earlier studies^16^,^44^,^49^. The new assay here developed responds to the effects of E1-phosphorylation on the assembly of the PDC, as reflected by the overall activity. Using the functional kinetic approach, we could assess the contribution of all lipoyl domain sources of the E2⋅E3BP core (L1, L2S, L1L2S, L3S’) on activation of the four PDK isoforms, while earlier studies were mostly focused on the interaction of L2 with PDKs^6^,^45^,^50^,^51^,^52^,^53^. Our recent findings suggested that the L1L2S tridomain gives rise to stronger, and with more points of interaction, with PDK1 and PDK2, than does the L2S didomain^48^. Additionally, the L3S’ didomain also revealed moderate interactions with both PDK1 and PDK2^48^.

The activation of the PDK isoforms (as reflected by the % PDC activity remaining) by the E2·E3BP and its derived domains is shown in Fig. 2 for PDK1 and PDK2, in Fig. 3 for PDK3 and in SI Fig. S2 for PDK4. The values of first order k_app_ of PDC inactivation by PDK1-PDK4 in the absence and presence of the E2· E3BP-derived lipoyl domains are presented in SI Table S1.

The data summarized in Fig. 4 provide striking evidence that of all four PDK isoforms, PDK2 is the only one that was able to significantly reduce PDC activity even in the absence of the E2·E3BP core or its derived domains (Fig. 2, bottom, Fig. 4, SI Table S1). The no change in inactivation resulted within experimental error from the presence of E2·E3BP core or its derived domains (Fig. 2, bottom, Fig. 4, SI Table S1). PDK2, apparently is also modestly activated by L3S’ with 6.5% PDC activity remaining, comparable to 5.7% observed with E2·E3BP core. Since PDK2 is the major isoform responsible for the regulation of PDC activity so far as wide tissue distribution, high level of expression and contribution to diabetes are concerned^7^,^42^,^49^,^54^, this is an important observation.

Unlike PDK2, the other three PDKs required activation by E2·E3BP core, in whose absence the PDC activity remaining was 93% for PDK1, 94% for PDK3 and 97% for PDK4 (see control experiments in Fig. 4 on the right-hand side of each panel).

The PDK1 was activated by any source of the inner lipoyl domain (L2) (i.e., E2⋅E3BP, L1L2S or L2S; see Fig. 2, top and Fig. 4). The activation of PDK1 by the L2 sources followed a specific pattern where L1L2S was most effective with 13% of PDC activity remaining. The overall pattern of activation was: L1L2S > E2⋅E3BP = L2S > L1 > L3S’, corresponding to PDC activities remaining of 13% (L1L2S), 28% (E2· E3BP), 29% (L2S), 62% (L1), and 83% (L3S’) in Fig. 4.

The PDK3 proved to be more difficult to activate, requiring a one hour pre-incubation with E2⋅E3BP core, or with its derived domains, before the E1 phosphorylation reaction is started, leading to some inconsistances in the literature^44^,^46^,^47^. As shown in Fig. 3, about 5% of the PDC activity remained when PDK3 was activated by E2·E3BP core (k_app_ of inactivation = 0.058 min^−1^), as compared with 94% activity remaining in the absence of activation (not presented). Our data correlate well with the 17-fold and 15-fold enhancement of PDK3 activity by E2 and E2·E3BP core, when the incorporation of ^32^P from [γ-^32^P]ATP into E1 was analyzed^44^. Much less PDK3 activation was achieved by other lipoyl domain sources according to PDC activity remaining: L1L2S (68%), L2S (95%), using the molar ratio for lipoyl domain source to PDK3 of 1:1 (Fig. 3, top). No further PDK3 activation was observed on increasing the amount of the lipoyl domain source, even at much higher molar ratios of lipoyl domain source to PDK3: L1L2S (23:1), PDK3:L2S (18:1), PDK3:L3S’ (33:1), and PDK3:L1 (350:1). Even under these conditions, 76.5% PDC activity remained with no additional PDC inactivation (Fig. 3, middle). These results suggest that PDK3 may require the catalytic domain from either E2 (**C**) or E3BP (**C’**) for activation, perhaps in combination with the lipoyl domain source. To test this hypothesis, we showed that while the independently expressed E2-catalytic domain (**C**) was ineffective in activating PDK3 by itself, in combination with L1L2S, only 2% of PDC activity remained after 20 min of incubation with a k_app_ of inactivation of 0.15 min^−1^ (Fig. 3, bottom). Since L1L2S by itself led to 68% of the PDC activity remained, the results suggested that a combination of **C** and L1L2S is needed for more effective activation of PDK3. This provides a remarkable example of synergistic catalysis by individual domains. When tested with PDK1, no such synergistic catalysis could be observed (data not shown).

Data for PDK4 presented in Fig. 4, in SI Fig. S2, and in SI Table S1 led to the following conclusions: (1) PDK4 was the least affected by interaction with lipoyl domain sources among the four PDK isoforms, irrespective of the lipoyl domain source, and even with prior pre-incubation. A modest reduction of the PDC activity, quoted as % of the PDC activity remaining, was observed only when E2⋅E3BP core (67%) or L3S’ (65%) were present, while L1L2S (92%), L2S (100%), and L1 (100%) were not effective despite their good binding constants to PDK4 presented for the first time below. Our results confirmed that PDK4 has the lowest activation by the E2·E3BP core among all four PDKs is in accord with reports from other groups^55^; (2) It was reported that PDK4 displayed the highest activity toward ^32^P incorporation into E1 in the absence of E2·E3BP core (basal activity) among all four PDKs which was not significantly changed in the presence of L2 or E2 ·E3BP core^55^. With highest basal activity reported in the literature, only a modest PDC inactivation by PDK4 was detected in our studies with no E2·E3BP core present (SI Fig. S2), reflecting the unique biochemical properties of PDK4. Earlier, the unique PDK4 structure with a favored open conformation of the active center has been reported. Based on structural data, the weaker binding affinity for ADP on comparison with ATP was suggested as resposible for a high basal activity of PDK4^55^,^56^. It is plausible that PDK4 may require factors other than PDC E2·E3BP core for activation to reach its full catalytic potential. Recently it was reported that PDK4 level in cardiac mitochondria is selectively regulated by the mitochondrial protease, Lon and its degradation by Lon depends on the energetic state of the mitochondria^57^.

### Specificity of interaction of PDK isozymes with E2·E3BP lipoyl domains monitored via site-specifically introduced external fluorophore

**Figure 1:**
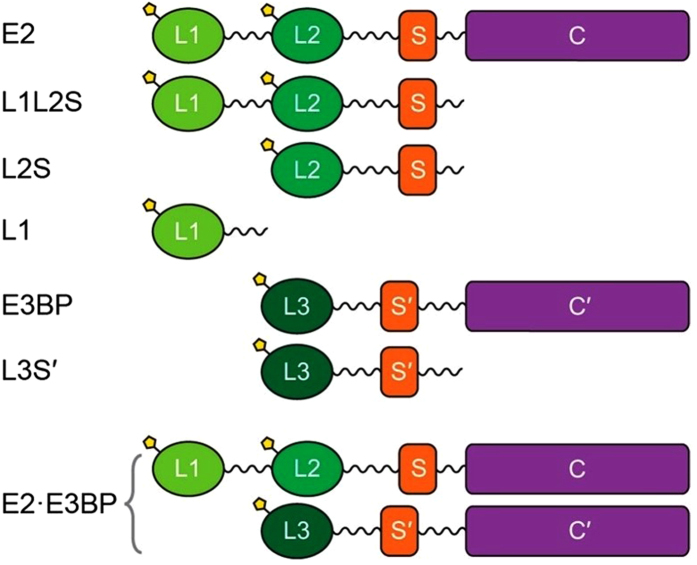
Schematic representation of the domains of the human pyruvate dehydrogenase E2⋅E3BP core.

**Figure 2:**
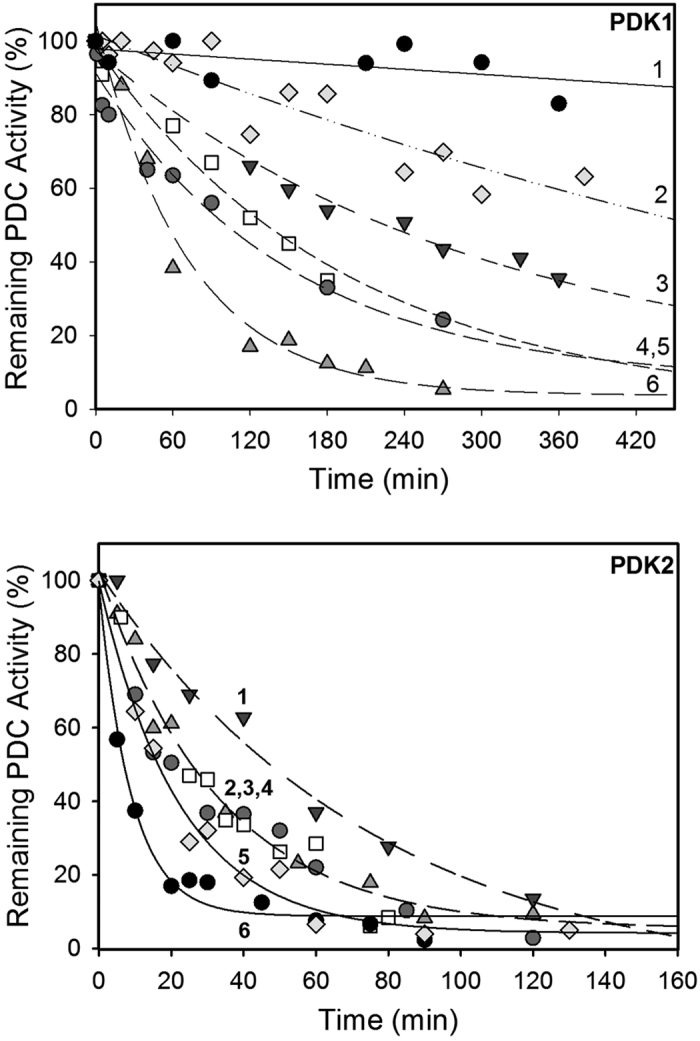
Time-dependence of PDC inactivation by PDK1 and PDK2. (**Top**) PDK1 (3.0 μg, 0.1 μM) in 50 mM KH_2_PO_4_ (pH 7.5) supplemented with 0.5 mM ThDP, 1.0 mM MgCl_2_, 4.0 mM DTT and 0.1 mM EDTA was incubated with E1 (75 μg, 1.95 μM) in the presence of either L1L2S (3.0 μg, 0.3 μM) (line 6, 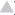); E2⋅E3BP (3.0 μg, 0.2 μM), (line 5, 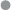); L2S (2.4 μg, 0.5 μM), (line 4, ◽); L1 (3.0 μg, 1.0 μM) (line 3, 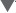); L3S’ (1.0 μg, 0.5 μM) (line 2, 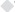) or with no lipoyl domain source (line 1, ●). Phosphorylation was initiated by ATP (0.5 mM) at 23 °C. (**Bottom**) PDK2 (6.6 μg, 0.48 μM) in 50 mM KH_2_PO_4_ (pH 7.5) supplemented with 0.5 mM ThDP, 1.0 mM MgCl_2_, 4.0 mM DTT, and 0.1 mM EDTA was incubated with E1 (80 μg, 3.5 μM) with no lipoyl domain source (line 6, ●); in the presence of either: L3S’ (1.0 μg, 0.24 μM) (line 5, 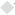); E2· E3BP (2.0 μg, 0.22 μM) (line 4, 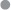); L1L2S (1.2 μg, 0.22 μM), (line 3, 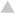); L2S (0.8 μg, 0.22 μM) (line 2, ◽) and L1 (0.4 μg, 0.22 μM), (line 1, 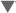). Phosphorylation was initiated by ATP (2.0 mM) at 30 °C. Aliquots (1 μg E1) were withdrawn at different times and were mixed with E2⋅E3BP and E3 at a mass ratio of E1:E2·E3BP: E3 of 1:3:3 to measure PDC activity.

**Figure 3:**
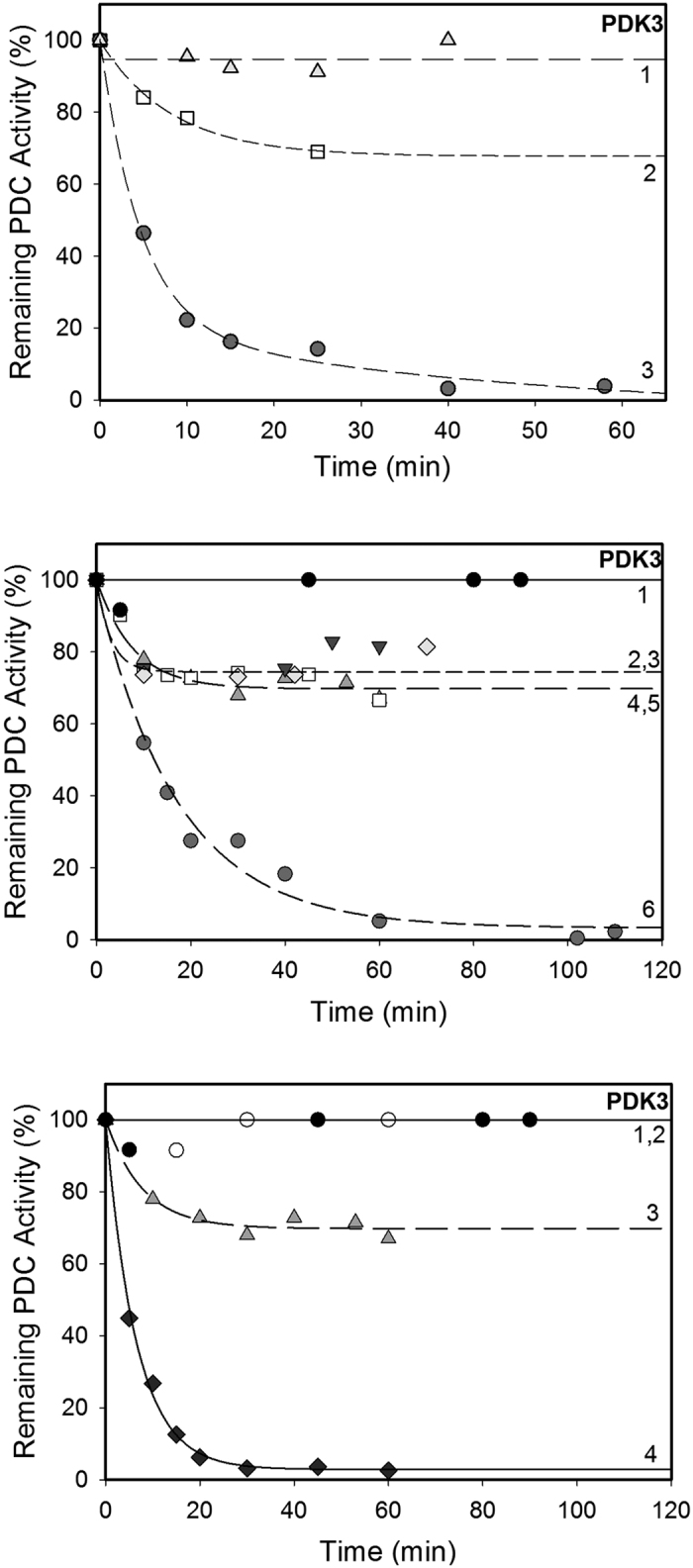
Time-dependence of PDC inactivation by PDK3. (**Top**) PDK3 (3.0 μg, 0.22 μM) in 20 mMTris·HCl (pH 7.4) supplemented with 0.1 M KCl, 5.0 mM MgCl_2_ and 2.0 mM DTT was incubated with E1 (80 μg, 3.5 μM) in the presence of either L2S (0.8 μg, 0.22 μM) (line 1, 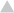); (line 2, ◽) L1L2S (1.2 μg, 0.22 μM) (line 2, ◽) or E2·E3BP (2.0 μg, 0.22 μM) (line 3, 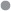). Phosphorylation was initiated by ATP (2.0 mM) at 30 °C. (**Middle**) PDK3 (1.0 μg, 0.12 μM) in 20 mMTris·HCl (pH 7.4) supplemented with 0.1 M KCl, 5.0 mM MgCl_2_ and 2.0 mM DTT was first pre-incubated for 1 hour at 4 °C with either E2·E3BP (45 μg, 7.55 μM), (line 6, 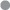); L1L2S (10 μg, 2.7 μM) (line 5, 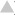); L2S (5.0 μg, 2.12 μM), (line 4, ◽); L1 (50 μg, 42 μM, (line 3, 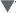); L3S’ (10 μg, 4.0 μM) (line 2, 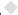) or with no source of the lipoyl domain (line 1, ●). Phosphorylation was initiated by ATP (0.1 mM) at 30 °C. (**Bottom**) PDK3 (1.0 μg, 0.12 μM) in 20 mMTris-HCl (pH 7.4) supplemented with 0.1 M KCl, 5.0 mM MgCl_2_ and 2.0 mM DTT was pre-incubated for 1 hour at 4 °C with either E2-Catalytic domain (1.0 μg, 0.33 μM) plus L1L2S (1.0 μg, 0.27 μM) (line 4, ♦); L1L2S (10 μg, 2.7 μM) (line 3, 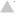); E2-Catalytic domain (45 μg, 4.8 μM) (2, ○) or with no lipoyl domain source (1, ●). Phosphorylation reaction was initiated by ATP (0.1 mM) at 30 °C.

**Figure 4:**
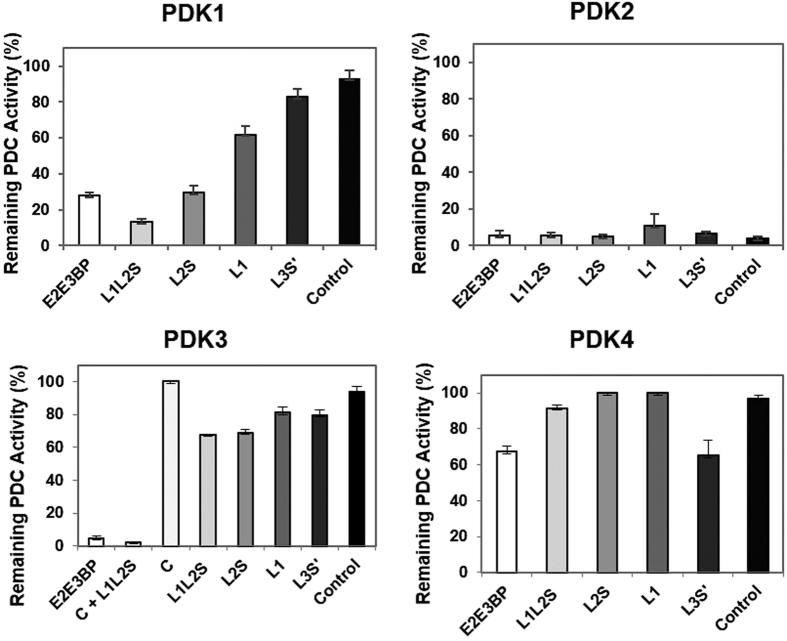
Comparison of the effect of the E2·E3BP core and its derived domains on PDC inactivation by PDK1-PDK4. Values on Y axis represent % of the remaining PDC activity. The E2·E3BP core and its C-terminally truncated proteins used for activation of PDK1- PDK4 are presented on the X axis. The time of E1 incubation with PDK1-PDK4 with or without lipoyl domain source was chosen as follows: 210 min for PDK1 (left panel, top); 90 min for PDK2 (right panel, top), and 60 min for PDK3 and PDK4 (left and right panels, bottom). Each experiment was done in triplicate and the standard error of mean (SEM) is shown.

In addition to a determination of the kinetics of inactivation of PDC by the PDK isoforms under the influence of the E2·E3BP core and its derived domains^44^,^57^, it was important to determine the binding constants between the PDK isoforms and the same E2·E3BP core and its derived domains. A fluorescence method was developed to introduce an external fluorophore specifically onto all E2·E3BP-derived lipoyl domains (L1, L2S, L1L2S-ML1, L1L2S-ML2, L3S’ in Fig. 1). In ML1 the lysine site of lipoylation in L1 is substituted to alanine, while in ML2, the lysine site of lipoylation in L2 is substituted to alanine so that there is only a single lipoylation site available for labeling. The following steps were used: (i) Complete lipoylation of the source of the lipoyl domain *in vitro* by a lipoyl ligase; (ii) Reduction of the E2·E3BP-derived lipoyl domains by tris(2-carboxyethyl)phosphine (TCEP); (iii) Attachment of the synthesized external fluorophore (4-((5-(dimethylamino)naphthalene)-1-sulfonamido)phenyl)arsenous acid (DANS-As) onto reduced lipoyl domains (see SI Fig. S3 for synthesis and SI Experimental Procedures). Mass spectrometry was used to monitor the consecutive reactions of lipoylation, reduction, then attachment of the external fluorophore and finally to determine the labeling efficiency as exemplified for L1. On reduction of the lipoamide tethered to the L1, its mass was shifted from 11,972.09 Da to 11,974.11 Da (the theoretical mass of the lipoylated L1 = 11,971.3 Da). After attaching the DANS-As, the L1 mass was shifted further from 11,974.11 Da to 12,372.09 Da corresponding to the mass of added DANS-As group. According to mass spectrometry, the labeling efficiency was 75%.

**Figure 5:**
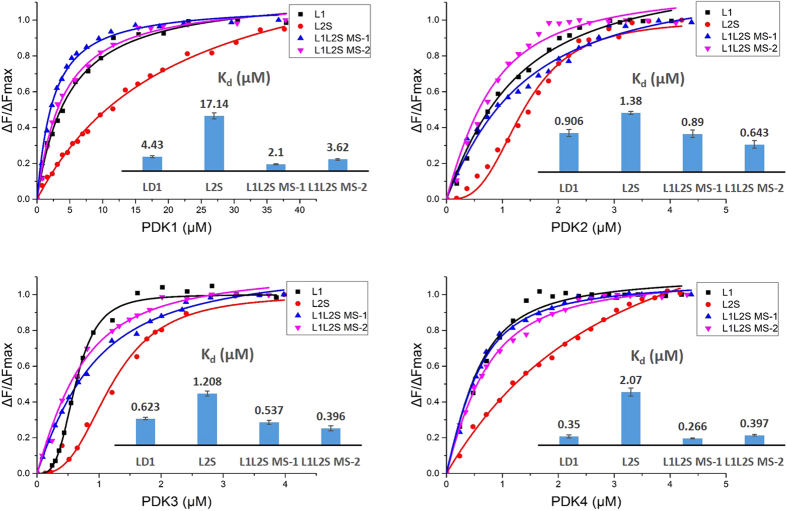
Fluorescence binding curves for DANS-As-labeled E2·E3BP derived lipoyl domains on titration by PDK isoforms. The DANS-As-labeled L1 (1.0 μM), or L2S (1.5 μM) or L1L2S MS-1 (2.5 μM) or L1L2S MS-2 (3.0 μM) in 30 mM KH_2_PO_4_ (pH 7.5) was titrated by PDK1 (0.78–37.93 μM) or PDK2 (0.18–4.38 μM) or PDK3 (0.24–3.86 μM) or PDK4 (0.24–4.2 μM) at 25 °C. The excitation wavelength was 338 nm and the emission spectra were recorded in the 425–600 nm range. In all cases the fluorescence intensity of the DANS-labeled lipoyl domains was enhanced on PDKs binding. For experimental details see SI Experimental procedures.

Typical changes in the fluorescence of the DANS-As-labeled L2S and DANS-As-labeled L3S’ on titration by PDK1 and PDK2 are shown in SI Fig. S4. It is evident that with the dansyl group attached to L2S (DANS-As-L2S), binding to PDK1 enhanced its fluorescence intensity. Similar fluorescence intensity changes were observed for DANS-As-labeled L1, L2S, L1L2S-ML1, L1L2S-ML2 on titration by each of the four PDK isozymes (Fig. 5). Attachment of the DANS-As group to L3S’, in contrast, led to DANS fluorescence quenching, rather than to enhancement on binding to PDK2 and PDK3 (SI Fig. S4, bottom presents data for PDK2). These data suggest different binding environment for DANS-As-L2S and DANS-As-L3S’ on binding the PDK isoforms, more hydrophobic environment (surrounded by protein) implied by the fluorescence enhancement, and more hydrophilic (more aqueous) environment implied by the fluorescence quenching^58^.

**Figure 6:**
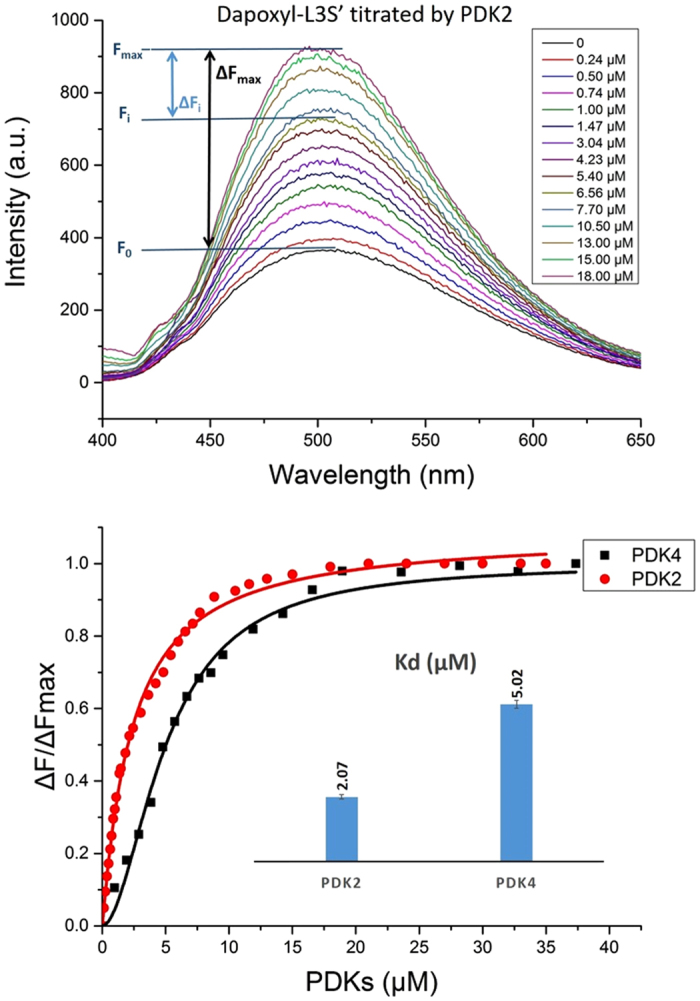
Binding of PDKs to dapoxyl-labeled L3S’. (Top) Fluorescence titration of dapoxyl-labeled L3S’ by PDK2. The dapoxyl-L3S’ (3 μM) in 30 mM KH_2_PO_4_ (pH 7.5) was titrated by PDK2 that was accompanied by the enhancement of the dapoxyl-L3S’ fluorescence intensity. The PDK4 behaves similarly. (Bottom) Fluorescence binding curves for dapoxyl-labeled L3S’ on titration by PDK2 and PDK4. Inset: the K_d_ values for PDK2-L3S’ and PDK4-L3S’ complexes. Excitation wavelength was at 327 nm and the emission spectra were recorded in the 400–650 nm range. No changes in fluorescence intensity of dapoxyl-L3S’ was observed on titration by PDK1 or PDK3.

A second fluorophore (the dapoxyl group) was also attached to L3S’, this time non-specifically to lysine residues of the L3, to confirm affinity of L3S’ to the PDK isoforms. As seen in Fig. 6 (top), fluorescence enhancement resulted from the interaction of dapoxyl-labeled L3S’ with PDK2 (Fig. 6, top displays data for PDK2). Similar fluorescence enchancement resulted from the interaction with PDK4 (not presented).

The fluorescence titration curves for DANS-As-labeled lipoyl domains on binding of PDK isoforms are summarized on Fig. 5 and the calculated values of K_d_ are presented in Table 1.

As reported in Table 1, among the four PDK isoforms, the interaction between PDK1 and different DANS-As-lebeled lipoyl domains is the weakest, which might explain why PDK1 is the only kinase that can phosphorylate all three sites of the E1α subunit^15^. It appears that PDK2 and PDK3 interact with the DANS-As-lebeled lipoyl domains similarly, with both DANS-As-L1 and DANS-As -L2S contributing significantly to binding. The K_d_ values of the PDK2-DANS-As-L1 complex (0.91 μM) and PDK2-DANS-As-L1L2S-ML1 complex (0.89 μM) are nearly identical, as are the values for the PDK3-DANS-As-L1 (0.62 μM) and PDK3-DANS-As-L1L2S-ML1 (0.54 μM) complexes, suggesting that the lipoamide region in L2 does not contribute to binding of PDK2 or PDK3. ThePDK4 has clear preference for L1 (K_d_,_L1_ = 0.35 μM; K_d_,_L2S_ = 2.1 μM), similarly to PDK2 and PDK3.

We also wished to investigate the interaction of the PDK isoforms with L3S’ labeled with both the DANS-As- and dapoxyl group. The PDK1 displayed no binding with either labeling, while PDK4 showed weak binding (Table 1). Analysis of the titration data indicated stronger binding to PDK2 (K_d_ = 2.1 μM for dapoxyl-L3S’, and 0.025 μM for DANS-As-L3S’) than to PDK4 (K_d_ ~ 5.0 μM with both labeled L3S’). The PDK3 isoform produced a signal only with L3S’.

In comparison with our data, an earlier report on the interaction of PDK4 with individual L1 and L2 domains was unable to identify these interactions by enthalpy changes using ITC and suggesting weak binding^55^. A K_d_ of 5 μM was reported for L3 binding to PDK4^55^, a value that correlates well with values of K_d_ = 5.93 μM (DANS-As-L3S’) and K_d_ = 5.02 μM (dapoxyl-L3S’) determined by fluorescence spectroscopy in Table 1. On the basis of the K_d_ values presented in Table 1, it became evident that L1 and L3S’ also participate in the interaction with some PDK isoforms, not recognized before. This conclusion is supported by a comparison of the K_d_ values in Table 1 with those reported earlier, and using different approaches to calculate binding constants. Values of K_d_ of ~175 μM (oxidized L2) and K_d_ ~ 130 μM (reduced L2) for the PDK2-L2 complex were obtained by analytical ultracentrifugation^59^. On conjugation of L2 to glutathione-S transferase, the PDK2-L2 complex appeared to be stronger with K_d_ ~ 3 μM (oxidized L2), and K_d_ ~ 0.4 μM (reduced L2), as compared with K_d_ ~ 22 μM (L1) and K_d_ ~ 35 μM (L3), where L1 and L3 were also conjugated to glutathione-S transferase^57^. A value of K_d_ ~ 10 μM was calculated for the PDK1-L2 and PDK2-L2 complexes using gel filtration chromatography, while the same method gave the following relative affinities for complexation with L2: PDK3 > PDK1 = PDK2 > PDK4^46^. A value of K_d_ = 1.17 ± 0.23 μM was obtained by ITC for the PDK3-L2 complex^47^.

### Site-specificity of the four PDK isoenzymes using single phosphorylation site E1 variants

As there are three phosphorylation sites in E1α (the E1 is an α_2_β_2_heterotetramer), it was also important to analyze with the newly developed assay the specificities of the four PDKs toward the three phosphorylation sites of E1. It was previously reported that all four PDKs phosphorylate *site 1* and *site 2*, but with different rates according to incorporation of ^32^P from [γ-^32^P]ATP into E1: for *site 1*, PDK2 > PDK4 ≈ PDK1 > PDK3; for *site 2*, PDK3 > PDK4 > PDK2 > PDK1. *Site 3* was phosphorylated by PDK1 only^16^,^17^,^52^. In this paper the specificity of the four PDKs toward the three phosphorylation sites was assessed by measuring the overall complex activity. The PDC was assembled with E1 variants containing double substitutions at phosphorylation sites, with only one site available for phosphorylation: E1-MS 2,3 (MS substituted at *sites 2* and *3, site* 1 is available); E1-MS 1,3 (MS substituted at *sites 1* and *3, site 2* is available); E1-MS 1,2 (substituted at *sites 1* and *2, site 3* is available) to test which of the three sites contributes the most to the inactivation of E1 by phosphorylation for each PDK.

With PDK1 the highest rate of PDC inactivation was toward the E1-MS 2,3 variant (*site* 1 available) (Table 2, SI Fig. S5). The remaining PDC activity was less than 10% after 50 min of incubation and k_app_ = 0.054 min^−1^ could be calculated for PDC inactivation. This rate was even higher than that for the wild-type E1 (k_app_ = 0.018 min^−1^). With the other two variants E1-MS 1,3 (*site 2* available) and E1-MS 1,2 (*site 3* available) no greater than 20% PDC inactivation resulted even after > 250 min of their treatment by PDK1, and did not allow calculation of rate constants for PDC inactivation in Table 2. The results in Table 2 are in agreement with the literature^16^,^17^.

With PDK2, *site 1* clearly contributed to PDC inactivation. After only 20 min, PDK2 was able to inactivate E1-MS 2,3 to less than 20% of PDC activity remaining with k_app_ of 0.061 min^−1^, comparable to the k_app_ of 0.057 min^−1^ for the wild-type E1 (Table 2). For the E1-MS 1,3 variant, however, nearly 100% of the PDC activity remained during the first 60 min of incubation with PDK2, and only after 120 min was PDK2 able to reduce PDC activity to ~50%. According to the reported data, the PDK2 displayed the highest activity for *site1* followed by PDK4 and PDK1^17^. Earlier, PDK3 was reported to have the lowest activity toward *site 1*, even when activated by the E2⋅E3BP core^16^,^17^, but was able to inactivate E1-MS 2,3 and E1-MS 1,3, or even wild-type E1 at rather similar high rates (0.099 min^−1^, 0.062 min^−1^ and 0.058 min^−1^, respectively). Again, *site 1* is mostly responsible for the PDC inactivation by PDK3, similarly to PDK1 and PDK2 (Table 2, SI Fig. S5). However, the highest activity exhibited toward *site2* makes PDK3 unique and differentiates it among the four PDKs. Although PDK4 could only partially inactivate PDC assembled with wild-type E1 (about 65% of the PDC activity remaining), its site-specificity was also examined. Most of the PDC activity lost was due to phosphorylation of site 1, since E1-MS 2,3 gave similar results to wild-type E1, where PDC activity remained at 70% or 65%, respectively, after 120 min (SI Fig. S5, bottom, right). On PDC reconstitution with E1-MS 1,3, the activity remained unchanged at 90–95% after 120 min. Again, the lack of significant PDC inactivation by PDK4 is consistent, irrespective of the site selected.

The following could be concluded: (i) Most of the PDC activity lost was due to phosphorylation at *site 1* on E1 when reacted with PDK1, PDK2, PDK3, and less so with PDK4. (ii) A high PDK3 activity toward *site 1* and *site 2* was detected indicating that E2·E3BP core is needed for its activation, as was confirmed by experiments with independently expressed E2 catalytic domain.

## Discussion

From these studies on activation of PDK 1–4 by the E2·E3BP core and its derived domains, it has become evident that each of the four PDK isoforms has its own preferences for activation by PDC, which are not limited to binding to the L2 or L3 domains only. The PDK1 was the only isoform that exhibits different activation by E2·E3BP derived domains (based on % of the PDC activity remaining) with L1L2S (k_app_ = 0.014 min^−1^) > E2·E3BP core (0.007 min^−1^) ≈ L2S (0.005 min ^−1^) > L1 (0.003 min ^−1^) > L3S’ (0.001 min^−1^) (SI Table S1). However, the k_app_ of PDC inactivation by PDK1 was still slow in comparison with PDK2. The order of PDK1 activation indicates best activation by the L1L2S tridomain; inclusion of L1 adds significantly to activation by L2S.

The activation of PDK2 by E2·E3BP core and its derived domains was more evident from the calculated k_app_ of PDC inactivation: it was the fastest rate among the four PDK isoforms even without activation (k_app_ ~ 0.11 min^−1^), a rate that was virtually unchanged within experimental error by E2·E3BP derived domains, with E2·E3BP core (k_app_ = 0.032 min^−1^) ≈ L1L2S (k_app_ = 0.031 min^−1^) ≈ L2S (0.028 min^−1^) > L1 (k_app_ = 0.013 min^−1^) (SI Table S1). Additionally, the PDK2 was also activated by L3S’ (k_app_ = 0.045 min^−1^), indicating very modest, if any, activation of PDK2 by E2·E3BP derived domains. But, binding was indeed observed between PDK2 and L3S’ according to fluorescence titration studies.

PDK3 required E2·E3BP core for its activation (k_app_ = 0.058 min^−1^), with essentially no activation by E2·E3BP derived domains. The E2 catalytic domain together with L1L2S tridomain provided the highest rate of PDC inactivation among all E2·E3BP-derived domains (k_app_ = 0.15 min^−1^), in what appears to be a case of synergistic catalysis by two domains.

PDK4 was only weakly activated by L3S’ (k_app_ = 0.008 min^−1^) and by E2·E3BP core (k_app_ = 0.007 min^−1^), but in both cases the rate of PDC inactivation was slow.

Further information was gathered from studying the single phosphorylation site E1 proteins. The loss of PDC activity with PDK1-3 was shown to be mostly due to phosphorylation of *site 1* with the following relative reactivities: PDK3 (k_app_ = 0.099 min^−1^) > PDK2 (k_app_ = 0.061 min^−1^) ≈ PDK1 (k_app_ = 0.054 min^−1^). With PDK3, the rate constant of PDC inactivation at phosphor-ylation *site 1* (k_app_ = 0.099 min^−1^) and *site 2* (k_app_ = 0.062 min^−1^) were nearly the same, unusual in this regard. For PDK4, the results raise a question: is this kinase really specific to PDC ? From these studies, it has also become evident that there is no strong correlation between the ability of the E2·E3BP-derived domains to activate PDK and their binding affinities for the PDK isoforms. Among the four PDK isoforms, PDK1 revealed the weakest binding of E2·E3BP-derived domains; its ability to bind any source of the lipoyl domain correlated well with low rates of PDC inactivation detected. The PDK1 isoform prefers binding to L1, rather than to L2; binding to L2 was very weak with K_d_ = 17 μM. Also, no apparent binding to L3S’ was detected for PDK1. In contrast, both PDK2 and PDK4 revealed binding to L3S’ with K_d_ = 2.07 μM and 5.02 μM, respectively, providing an explanation why they could be activated by L3S’. It appears that PDK2 and PDK3 interact similarly with the lipoyl domain source, with both L1 and L2S contributing significantly to binding, a finding not correlated with their ability to activate the kinases. As mentioned above, PDK2 is active even in the absence of the E2·E3BP core or its derived domain, while only the E2·E3BP core could activate PDK3. This disagreement between binding and activation abilities of E2·E3BP derived domains was even more pronounced with PDK4, where with good binding constants to the lipoyl domain source, they could not achieve activation of PDK4.

We can draw the following general conclusions of importance relevant to PDK isozyme-specific drug design.

1. No strong correlation could be observed between the ability of E2⋅E3BP core and its derived domains to activate the PDK isoforms and the binding constants for the corresponding complexes formed between a PDK and an E2⋅E3BP-derived domain.
2. The approach to inhibit interaction of PDK isoforms and E2⋅E3BP core as a route to intervention in diseases is not promising for PDK2 (no activation is needed, only modest additional activation/inhibition results from the presence of E2⋅E3BP-derived domains), or PDK4 (no significant activation results from E2⋅E3BP core or from any of the E2⋅E3BP-derived domains. On the other hand, the approach is plausible and promising for PDK1 and PDK3.
3. PDK3 alone among the four isoforms is subject to activation by a combination of L1L2S and the E2 catalytic domain, providing a novel hitherto unexplored target for drug design.

## Additional Information

**How to cite this article:** Guevara, E. L. *et al*. Global view of cognate kinase activation by the human pyruvate dehydrogenase complex. *Sci. Rep.* **7**, 42760; doi: 10.1038/srep42760 (2017).

**Publisher's note:** Springer Nature remains neutral with regard to jurisdictional claims in published maps and institutional affiliations.

## Supplementary Material

Supplementary Information

## Figures and Tables

**Table 1:** K_d_ values for binding of the E2·E3BP-derived domains to PDK1-PDK4 as detected by fluorescence spectroscopy.

Kd (μM)	DANS-L1	DANS-L2S	DANS-L1L2S MS1	DANS-L1L2S MS2	DANS-L3S’	Dapoxyl-L3S’
PDK1	4.43 ± 0.35	17.14 ± 0.94	2.10 ± 0.12	3.62 ± 0.25	No binding	No binding
PDK2	0.91 ± 0.08	1.38 ± 0.03	0.89 ± 0.09	0.64 ± 0.09	0.025^a^	2.07 ± 0.06
PDK3	0.62 ± 0.03	1.21 ± 0.06	0.54 ± 0.05	0.396 ± 0.06	0.103^a^	No binding
PDK4	0.35 ± 0.06	2.07 ± 0.16	0.27 ± 0.02	0.397 ± 0.03	5.93 ± 1.06	5.02 ± 0.13

^a^On binding of PDK2 and PDK3 to DANS-L3S’, quenching of the DANS-L3S’ fluorescence was observed while in others enhancement resulted. See SI Experimental Procedures for details.

**Table 2:** Kinetic parameters for E1 and its doubly substituted (single phosphorylation site) variants inactivated by PDK1- PDK3.

k_app_ (min^−1^)	E1	E1-MS2,3	E1-MS1,3	E1-MS1,2
PDK1^a^	0.018 ± 0.003	0.054 ± 0.025	n/a^b^	n/a^b^
PDK2^a^	0.057 ± 0.011	0.061 ± 0.006	n/a^c^	n/a
PDK3^a^	0.058 ± 0.016	0.099 ± 0.032	0.062 ± 0.01	n/a

^a^PDK1 and PDK2 were activated by L1L2S; PDK3 was activated by E2·E3BP core.

^b^n/a, not available. No greater than 20% of E1-MS1,3 and E1-MS1,2 inhibition by PDK1 was detected.

^c^About 100% activity remained after 60 min treatment by PDK2.

^a,b,c^The experimental conditions are presented on SI Fig. S5 legend. Time course of the fraction of the remaining activity was fit to single exponential according to eq. f = f_1_ × (1 − e^−kt^).
