# Supplementary material for: Global view of cognate kinase activation by the human pyruvate dehydrogenase complex
Source: Sci Rep. 2017 Feb 23;7:42760. doi: 10.1038/srep42760 (PMC5322387; doi:10.1038/srep42760)
Supplement: Supplementary Information [file srep42760-s1.pdf]

SUPPLEMENTARY INFORMATION

Global view of cognate kinase activation by the human pyruvate dehydrogenase complex

[&+]**Elena L. Guevara,** [&+]**Luying Yang,** [‡]**Barbara Birkaya,** [&]**Jieyu Zhou,** [&]**Natalia S. Nemeria,** [‡*]**Mulchand S. Patel and** [&*]**Frank Jordan**[#]

From the [&]Department of Chemistry, Rutgers, The State University of New Jersey, Newark, New Jersey 07102 and [‡]Department of Biochemistry, Jacob School of Medicine and Biomedical Sciences, University at Buffalo, The State University of New York, Buffalo, New York 14214

SI, EXPERIMENTAL PROCEDURES

*Materials***.** Ni-NTA agarose used for protein purification was purchased from GE Healthcare Life Sciences. Thiamin diphosphate (ThDP), dithiothreitol (DTT), isopropyl-β-D-thiogalactopyranoside (IPTG), NAD^+ and coenzyme A (CoA) were purchased from Affymetrix USB. Sodium pyruvate was purchased from Sigma Aldrich.

Synthesis of (4-((5-(dimethylamino)naphthalene)-1-sulfonamido)phenyl)arsenous acid (DANS-As). The synthesis of DANS-As is presented in Figure S3. Briefly, p-arsanilic acid (10.85g, 50 mmol) was acidified by HCl and then reduced to (4-aminophenyl) arsenous acid using sulfur dioxide with sodium iodide as catalyst[1-3]. The (4-aminophenyl) arsenous acid (149 mg, 0.742 mmol) was then coupled to dansyl chloride (100 mg, 0.371 mmol) at pH 10 for 8–12 h at room temperature to produce (4-((5-(dimethylamino)naphthalene)-1-sulfonamido) phenyl)arsenous acid (DANS-As).

Bacterial Strains, Plasmids, Overexpression, and Purification. Recombinant wild-type E1 was overexpressed in *E. coli* BL21(DE3) cells harboring pET-28b-E1α/E1β (a co-expression vector with coding sequences of both E1α and E1β subunits) and was purified using a Ni-NTA-agarose column as described previously[4,5]. Several doubly substituted (single phosphorylation site) E1α variants were used in this study: E1-Ser271Ala /Ser203Ala (serine residues at phosphorylation *site 2* and *site 3* replaced by alanine and only serine at *site 1* available for phosphorylation); E1-Ser264Ala/ Ser203Ala (*site 2* available) and E1-Ser264Ala/Ser271Ala (*site 3* available)[6-8].

Recombinant E2·E3BP core was overexpressed in *E. coli* cells and was purified as described previously[9]. Construction of the plasmids for the following recombinant E2•E3BP-derived domains used in this study had been reported elsewhere: L1, containing the outer lipoyl domain and linker (residues 1−98); the L2S didomain containing the inner lipoyl domain (L2), second

hinge region, peripheral subunit-binding domain (S), and third hinge region (residues 128−330); the L1L2S tridomain, which comprises L1, L2, both hinge regions, and the subunit-binding domain (S, residues 1−330); the L3S' didomain of E3BP (residues 1−230), all expressed from pET28b in *E. coli* BL21 (DE3) cells, and purified using Ni Sepharose 6 Fast Flow column[10]. Two singly substituted variants of L1L2S were also used in this study: L1L2S-ML1 with the Lys46Ala substitution in the outer L1 domain, hence the inner L2 domain available for lipoylation, and L1L2S-ML2 (Lys173Ala substitution in the inner L2 domain, hence the outer L1 domain available for lipoylation). Recombinant E3 was overexpressed in *E. coli* cells and was purified as described earlier[11].

Recombinant rat PDK1, rat PDK2, human PDK3 and rat PDK4 were overexpressed and purified individually in *E. coli* BL21(DE3) cells transformed with pPDK expression vector using protocols reported in the literature[6,12-14]. According to the literature, the amino acid sequence of human PDK1 has 93% identity with rat PDK1, while human PDK2 has 96% identity with rat PDK2[15]. Human PDK3 shares 68% and 67% identity with human PDK1 and human PDK2, respectively[15].

Construction of Plasmid and Expression and Purification of the E2 Catalytic Domain. For expression of the E2 catalytic domain (**C**), DNA encoding residues 305-561 in wild-type human E2 comprising catalytic domain and linker region in front of it was synthesized by DNA2.0, Inc. (Menlo Park, CA). The E2CD gene was optimized for expression in *E. coli* cells and was inserted into pET-22b (+) through the BamHI and XhoI restriction sites. The TEV cleavage site was introduced in front of the XhoI site and the resulting plasmid was expressed in BL21 (DE3) cells. Cells were grown in LB medium supplemented with 50 μg/mL ampicillin and protein expression was induced by 0.50 mM isopropyl 1-thio-β-D-galactopyranoside (IPTG) overnight

at 30 ^{o}C. Cells were resuspended in 50 mM KH_2PO_4 (pH 7.5) containing 0.30 M KCl, 1 mM DTT, 25 mM imidazole and protease inhibitor cocktail tablets (Roche Diagnostics, GmbH, Germany). Cells were treated with lysozyme (0.6 mg/mL) and then by 1,000 units of DNase1 (NEB) and 1,000 units of micrococcal nuclease (NEB) to remove nucleic acids. The protein was purified using a Ni Sepharose 6 Fast Flow column (GE Healthcare) with 50 mM KH_2PO_4 (pH 7.5) containing 0.30 M KCl and 25 mM imidazole as binding buffer and 50 mM KH_2PO_4 (pH 7.5) containing 0.30 M KCl and 50 mM imidazole as washing buffer. Protein was eluted with 350 mM imidazole in binding buffer and was stored in 50 mM KH_2PO_4 (pH 7.5) containing 0.4 M NH_4Cl, 0.5 mM EDTA, 1.0 mM DTT and 1.0 mM benzamidine.HCl at -80 ^{o}C.

In vitro lipoylation of the E2·E3BP-derived domains. The E2·E3BP-derived domains were lipoylated *in vitro* using *E. coli* lipoyl protein ligase as reported by Rutgers[16]. Lipoylation was confirmed by FT-MS using the electrospray ionization sampling method.

Reaction of DANS-As with the Source of the Lipoyl Domains (L1, L2S, L1L2S, L1L2S-ML1, L1L2S-ML2 and L3S'). First, the lipoylated source of the lipoyl domain (50 μM) in 30 mM KH_2PO_4 (pH 7.5) containing 0.15 M NaCl (sample buffer) was reduced by incubation for 5 min with TCEP (100 μM) at room temperature, resulting in fully reduced lipoyl domains according to FT-MS. Second, the reduced lipoyl domains were reacted with 150 μM DANS-As for 2 h at room temperature, leading to chemically modified lipoyl domains. The excess of TCEP and DANS-As were removed from the reaction mixture with a 10K MWCO centrifugal filter unit (Vivaspin 500, 10K MWCO). Modification of the lipoyl domains was confirmed by FT-MS.

Enzyme Activity Measurements. Overall PDC activity was measured by assembly of E1 with recombinant E2·E3BP core and recombinant E3. The E1 component (1.0 μg) was preincubated in the cuvette with a mixture of E2·E3BP core and E3 at a mass ratio of 1:3:3 for 1 min at 37 ^{o}C

in 50 mM KH_2PO_4 (pH 7.5) containing 2 mM $MgCl_2$, 0.20 mM ThDP, 4.0 mM DTT and 2.0 mM NAD^+. The reaction was initiated by the addition of 2 mM pyruvate and 0.20 mM CoA and the formation of NADH (H^+) was monitored at 340 nm at 37 °C[6].

Phosphorylation of E1 and its variants by PDK isozymes. The E1 and its E1-MS 2,3 (*site1* available), E1-MS 1,3 (*site 2* available), and E1-MS 1,2 (*site 3* available) variants were phosphorylated by the four PDK isoforms in the presence of E2·E3BP core or the E2•E3BP-derived proteins (0.2-50 μM), or in their absence. A mass ratio of E1: PDK isoforms of 25: 1 was used. Phosphorylation by PDK1 and by PDK2 was performed in 50 mM KH_2PO_4 (pH 7.5) containing 0.5 mM ThDP, 1.0 mM $MgCl_2$, 4.0 mM DTT and 0.1 mM EDTA at 30 °C[6]. For PDK3 and PDK4, the phosphorylation reaction was preceded by their activation in the presence of the source of the lipoyl domain for 1 h at 4 °C as reported in the literature[12]. Phosphorylation by PDK3 and PDK4 was performed in 20 mM Tris-HCl (pH 7.4) containing 5.0 mM $MgCl_2$, 0.1 M KCl, and 2.0 mM DTT on the basis of a previous report[12]. The phosphorylation by PDK4 was also performed in KH_2PO_4 (pH 7.5). The phosphorylation reaction was initiated by addition of 0.5 mM ATP (PDK1 and PDK4) or 2.0 mM ATP (PDK2, PDK3) or 0.1 mM ATP (PDK3). Aliquots (1 μg of E1) were withdrawn at the indicated times and added to a 1 mL cuvette containing all components for PDC assay. PDC was allowed to assemble for 1 min at 37 °C and the reaction was started by addition of pyruvate and CoA as above.

Fluorescence Spectroscopy. For the fluorescence titration of the DANS-AS-labeled lipoyl domains by PDK isozymes, the DANS-As-modified lipoyl domain (2 μM) or dapoxyl-labeled L3S' (2 μM) in 30 mM KH_2PO_4 (pH 7.5) were titrated by PDK1 (0.78-37.93 μM), or PDK2 (0.18-4.38 μM), or PDK3 (0.24-3.86 μM), or PDK4 (0.24-4.20 μM). Fluorescence spectra were recorded at 25 °C using a Cary Eclipse spectrometer. The excitation wavelength was 338 nm for

DANS-As-labeled E2·E3BP-derived lipoyl domains and 327 nm for dapoxyl-lebeled-L3S' and the emission spectra were recorded in the range of 400-650 nm in 3 mL quartz cuvettes. Data were fitted to a Hill equation 2,

$$\Delta F / \Delta F_{max} = [PDK]^n / (S_{0.5}^n + [PDK]^n) \qquad (2)$$

where $\Delta F / \Delta F_{max}$ is a relative fluorescence; $\Delta F = F_{max} - F_i$, where F_{max} is a maximum fluorescence intensity reached on titration by PDK and F_i is a fluorescence intensity at a given concentration of PDK; $\Delta F_{max} = F_{max} - F_o$ where F_o is the initial fluorescence before addition of PDK; $S_{0.5}$, is the concentration at half saturation. *n* is the Hill coefficient. For n=1, the value of $S_{0.5}$ is equal to K_d.

SI REFERENCES

1. Stevenson, K. J., Hale, G. & Perham, R. N. Inhibition of pyruvatedehydrogenase multienzyme complex from *Escherichia coli* with mono- and bifunctional arsenoxides. *Biochemistry*, **17,** 2189-2192 (1978).
2. Adamson, S. R. & Stevenson, K. J. Inhibition of pyruvatedehydrogenasemultienzyme complex from *Escherichia coli* with a bifunctionalarsenoxide:selective inactivation of lipoamide dehydrogenase. *Biochemistry*, **20**, 3418-3424 (1981).
3. Adamson, S. R., Robinson, J. A. & Stevenson, K. J. Inhibition of pyruvate dehydrogenase multienzyme complex from *Escherichia coli* with radiolabeled bifunctional arsenoxide: evidence for an essential histidine residue at the active site of lipoamide dehydrogenase. *Biochemistry*, **23**, 1269-1274 (1984).
4. Korotchkina, L.G., Sidhu, M. S. & Patel, M.S. Characterization of testis-specific isoenzymes of human pyruvatedehydrogenase. *J. Biol. Chem*. **281**, 9688-9696 (2006).

5. Seifert, F., *et al.*, Direct kinetic evidence for half-of-the-sites reactivity in E1 component of the human pyruvatedehydrogenase complex through alternating sites cofactor activation. *Biochemistry*, **45**, 12775-12785 (2006).

6. Korotchkina, L. G. & Patel, M. S. Probing the mechanism of inactivation of human pyruvate dehydrogenase by phosphorylation of three sites. *J. Biol. Chem*. **276,** 5731-5738 (2001).

7. Korotchkina, L. G. & Patel, M. S. Site specificity of four pyruvate dehydrogenase kinase isoenzymes toward the three phosphorylation sites of human pyruvate dehydrogenase. *J. Biol. Chem*. **276**, 37223-37229 (2001).

8. Korotchkina, L.G., & Patel, M. Mutagenesis studies of the phosphorylation sites of recombinant pyruvate dehydrogenase. Site-specific regulation. *J. Biol. Chem*. **270**, 14297-14304 (1995).

9. Hiromasa, Y., Fujisawa, T., Aso, Y. & Roche, T. E. Organization of the cores of the mammalian pyruvate dehydrogenase complex formed by E2 and E2 plus the E3-binding protein and capacities to bind E1 and E3 components. *J. Biol. Chem*. **279**, 6921-6933 (2004).

10. Patel, M.S., Korotchkina, L.G. & Sidhu, S. Interaction of E1 and E3 components with the core proteins of the human pyruvate dehydrogenase complex. *J. Mol. Catal. B.Enzym.***61**, 2-6 (2009).

11. Liu, T.C., Hong, Y.S., Korotchkina, L.G., Vettakkorumakankav, N. N. & Patel, M.S. Site-directed mutagenesis of human dihydrolipoamide dehydrogenase: role of lysine-54 and glutamate-192 in stabilizing the thiolate-FAD intermediate. *Protein Express.Purif.* **16**, 27-39 (1999).

12. Baker, J. C., Yan, X., Peng, T., Kasten, S. & Roche, T. Marked differences between two

isoforms of human pyruvate dehydrogenase kinase. *J. Biol. Chem*. **275**, 15773-15781 (2000).

13. Bowker-Kinley, M. M., Davis, W. I., Wu, P., Harris, R. A. & Popov, K. M. Evidence for existence of tissue-specific regulation of the mammalian pyruvate dehydrogenase complex. *Biochem. J.,* **329**, 191-196 (1998).

14. Wynn, R. M., *et al.* Pyruvate dehydrogenase kinase-4 structures reveal a metastable open conformation fostering robust core-free basal activity. *J. Biol. Chem*. **283**, 25305-25315 (2008).

15. Gudi, R., Bowker-Kinley, M. M., Kedishvili, N. Y., Zhao, Y. & Popov, K. M. Diversity of the pyruvate dehydrogenase kinase gene family in humans, *J. Biol. Chem.***270**, 28989-28994 (1995).

16. Balakrishnan, A., Nemeria, N. S., Chakraborty, S., Kakalis, L. & Jordan, F. Determination of pre-steady-state rate constants on the *Escherichia coli* pyruvate dehydrogenase complex revealed that loop movement controls the rate-limiting step. *J.Amer.Chem.Soc.***134**, 18644-18655 (2012).

SI Figure Legends

Figure S1 - ThDP-bound and lipoamide-bound covalent intermediates on the PDC pathway.

Figure S2. Time-dependence of PDC inactivation by PDK4. (**Top**) PDK4 (2.0 μg, 0.43μM) in 20 mM KH_2PO_4 (pH 7.5) supplemented with 2.0 mM $MgCl_2$, 0.2 mM EDTA and 2.0 mM DTT was pre-incubated for 1 h at 4 °C with E2·E3BP (120 μg, 40 μM) (line 2, ●), or in its absence (line 1,●). E1 (77 μg, 10 μM) was then added and phosphorylation was initiated by ATP (0.5 mM) at 23 °C. (**Bottom**) PDK4 (2.0 μg, 0.22 μM) in 20 mM Tris·HCl (pH 7.4) supplemented with 0.1 M KCl, 5.0 mM $MgCl_2$, 2.0 mM DTT was pre-incubated for 1 h at 4 °C with either E2·E3BP (238 μg, 40 μM), (line 6, ●); L3S'(125 μg, 50 μM), (line 5,◆); L1L2S (37 μg, 10 μM), (line 4, ▲), L2S (12 μg, 5.0 μM (line 3, □), L1 (12 μg, 10 μM) (line 2,▼), or with no source of the lipoyl domain (line 1, ●) Phosphorylation was initiated by ATP (0.5 mM) at 23 °C.

Figure S3. Synthesis of (4-((5-(dimethylamino)naphthalene)-1-sulfonamido)phenyl)arsenous acid (DANS-As).

Figure S4. Fluorescence titration of DANS-As labeled L2S and DANS-As-labeled L3S' by PDK2. (Top) Enhancement of fluorescence of the DANS-As-L2S on PDK2 binding. DANS-As-L2S (1.5 μM) in 30 mM KH_2PO_4 (pH7.5) was titrated by PDK2 (3.1-59.1 μM). (Bottom) Quenching of the fluorescence of DANS-L3S' on PDK2 binding. DANS-L3S' (1.5 μM) in 30 mM KH_2PO_4 (pH 7.5) was titrated by PDK2 (0.09-1.60 μM).

Figure S5. Time-dependence of PDC inactivation by PDK1-PDK4 using single phosphorylation site (doubly-substituted) E1variants. **Top**, left:The E1 (□)or E1-MS 2,3 (▲) or E1-MS 1,3 (◆)or E1-MS 1,2(■), all at concentration of 5.0 μM (38.5 μg), were incubated with PDK1 (0.3 μM, 1.54 μg) activated by L1L2S (0.8 μM, 1.54 μg) in 50 mM KH_2PO_4 (pH 7.5). **Top**, right: The E1 (□) or E1-MS 2,3 (▲) or E1-MS 1,3 (◆) or E1-MS 1,2(■), all at 3.5 μM (81 μg) were incubated

with PDK2 (0.48 µM, 6.6 µg) in 50 mM KH_2PO_4 (pH 7.5). **Bottom**, left: PDK3 (0.12 µM, 1.0 µg) was first activated by E2•E3BP (7.55 µM, 45 µg) in 20 mM Tris·HCl (pH 7.4). The E1 (□) or E1-MS 2,3 (▲) or E1-MS 1,3 (◆) or E1-MS 1,2(■), all at concentration of 1.95 µM, (3.0 µg) was then added to start phosphorylation. **Bottom**, right: PDK4 (0.22 µM, 2.0 µg) was first activated by E2•E3BP (40 µM, 238 µg) in 20 mM Tris·HCl (pH 7.4). The E1 (□) or E1-MS 2,3 (▲) or E1-MS 1,3 (◆)or E1-MS 1,2 (■), all at concentration of 3.5 µM, (54 µg) was then added to start the phosphorylation. Time course of the fraction of the remaining activity was fit to a single exponential.

Mechanism of pyruvate dehydrogenase complex with role of ThDP and lipoic acid.

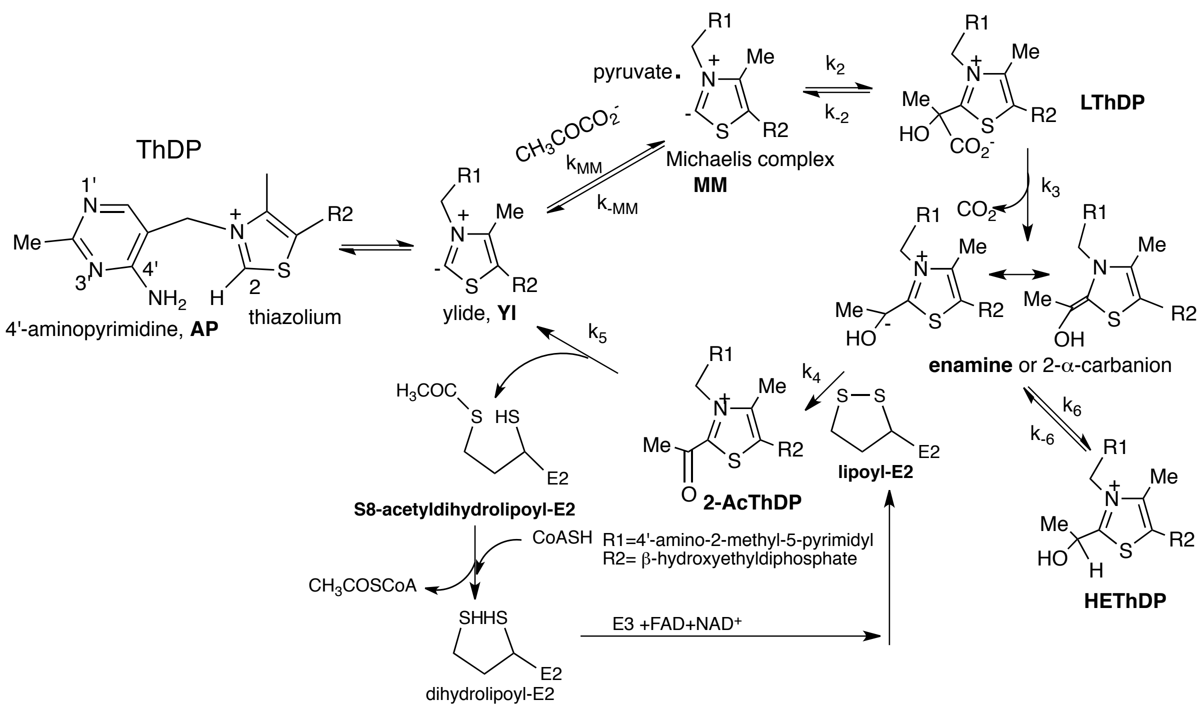

Figure S1.

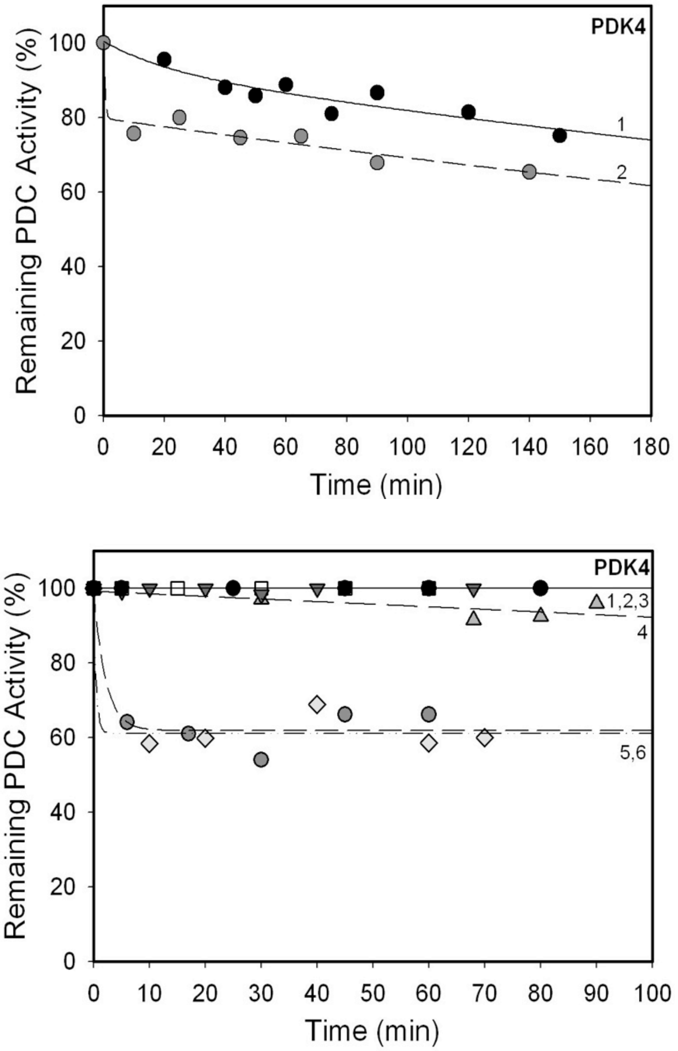
PDK4
Remaining PDC Activity (%)
Time (min)
1
2
PDK4
Remaining PDC Activity (%)
Time (min)
1,2,3
4
5,6

Figure S2.

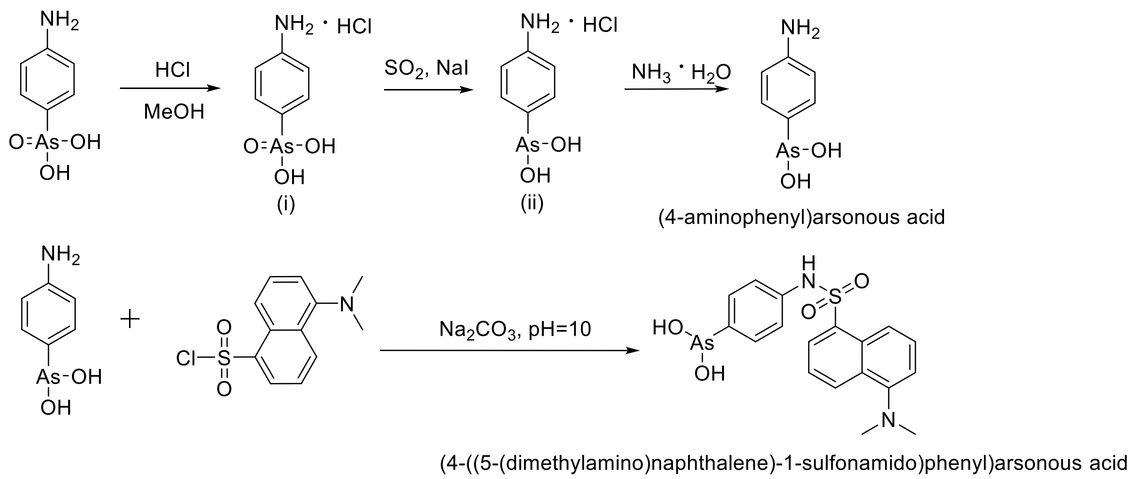
NH2
HCl
MeOH
O=As-OH
OH
NH2 · HCl
(i)
SO2, NaI
(ii)
NH3 · H2O
As-OH
(4-aminophenyl)arsonous acid
Cl
Na2CO3, pH=10
HO
As
N
S
(4-((5-(dimethylamino)naphthalene)-1-sulfonamido)phenyl)arsonous acid

Figure S3.

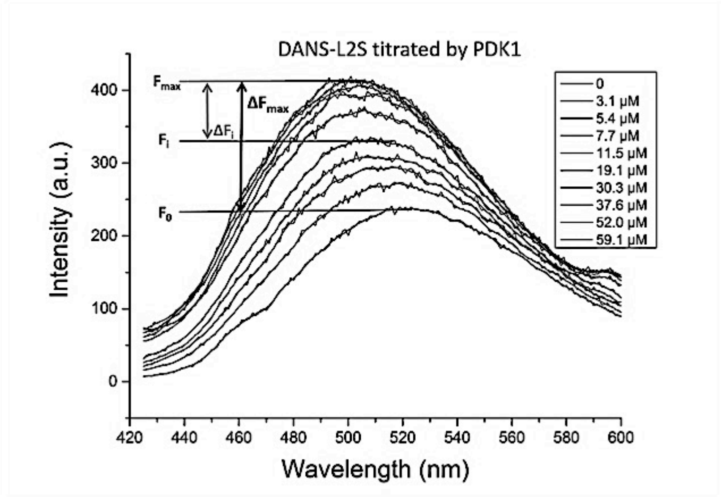
DANS-L2S titrated by PDK1
Intensity (a.u.)
Wavelength (nm)
F_max
ΔF_max
ΔF_i
F_i
F_0
0
3.1 μM
5.4 μM
7.7 μM
11.5 μM
19.1 μM
30.3 μM
37.6 μM
52.0 μM
59.1 μM

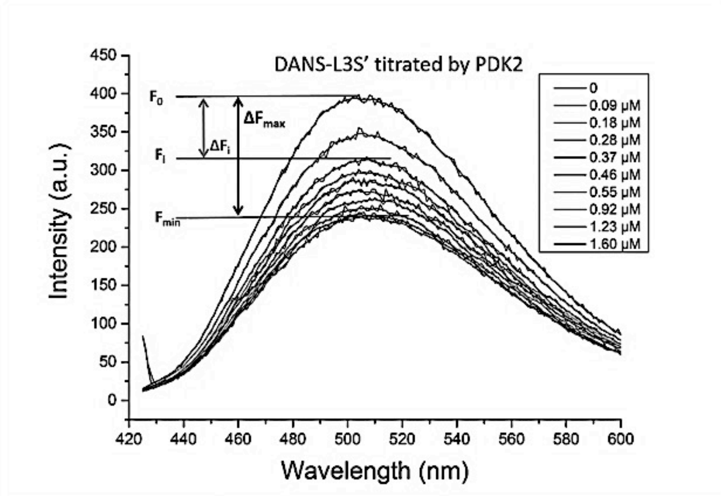
DANS-L3S' titrated by PDK2
Intensity (a.u.)
Wavelength (nm)
F_0
ΔF_max
ΔF_i
F_i
F_min
0
0.09 μM
0.18 μM
0.28 μM
0.37 μM
0.46 μM
0.55 μM
0.92 μM
1.23 μM
1.60 μM

Figure S4.

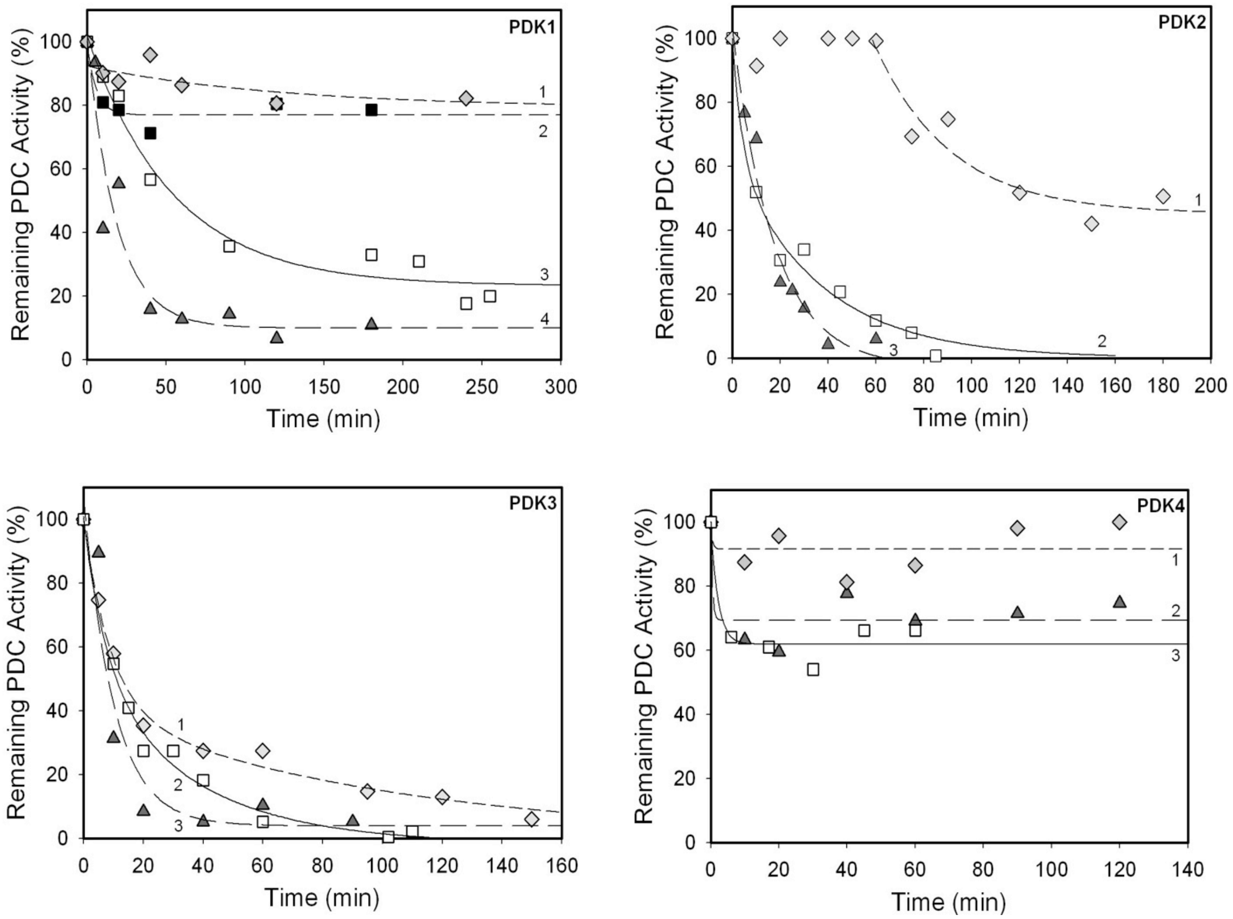
PDK1
PDK2
PDK3
PDK4
Remaining PDC Activity (%)
Time (min)
1
2
3
4
0
20
40
60
80
100
50
150
200
250
300
120
140
160
180

Figure S5.

Table S1. PDC inactivation by PDK1-PDK4 without and with lipoyl domain source[a,b,c].

PDK isoforms	k_{app} of PDC inactivation without and with lipoyl domain source (min^{-1})							
	none	L1	L2S	L1L2S	C	C + L1L2S	L3S'	E2·E3BP
PDK1	n/a	0.003 (204)	0.005 (139)	0.014 (50)	0.003 (231)	0.007 (99)	0.001 (630)	0.007 (99)
PDK2	0.11 (6)	0.013 (53)	0.028 (25)	0.031 (22)	0.12 (6)	0.10 (7)	0.045 (15)	0.032 (22)
PDK3	n/a	0.004 (n/a)	0.006 (n/a)	0.012 (n/a)	n/a	0.15 (5)	0.003 (n/a)	0.058 (12)
PDK4	n/a	n/a	n/a	n/a	n/a	n/a	0.008 (n/a)	0.007 (n/a)

[a] The half time of PDC inactivation ($t_{1/2,\ min}$) is presented in the parentheses. [b]Time course of the fraction of the remaining PDC activity was fit to a single exponential. [c]n/a, data not available because no inactivation was detected.
